# Job vacancies at the European Centre for Disease Prevention and Control

**DOI:** 10.2807/1560-7917.ES.2018.23.43.1810251

**Published:** 2018-10-25

**Authors:** Eurosurveillance editorial team

**Affiliations:** 1 European Centre for Disease Prevention and Control (ECDC), Stockholm, Sweden

The European Centre for Disease Prevention and Control (ECDC) invites applications for the following positions:

**Head of Section European and International Cooperation**
The application deadline expires on 26 Nov 2018.
 
**International Relations Officer**
The application deadline expires on 22 Nov 2018.

 

For more information, please visit the ECDC website: 

https://ecdc.europa.eu/en/work-us/vacancies?f%5B0%5D=deadline_date%3A1

